# Beliefs, screening attitudes and breast cancer awareness of young women with neurofibromatosis type 1: A reflexive thematic analysis

**DOI:** 10.1177/13591053241255053

**Published:** 2024-06-10

**Authors:** Caitríona Plunkett, Melissa Pilkington

**Affiliations:** Manchester Metropolitan University, UK

**Keywords:** breast awareness intervention, breast cancer awareness, neurofibromatosis type 1, qualitative methods, rare disease research, reflexive thematic analysis

## Abstract

Neurofibromatosis type 1 (NF1) predisposes individuals to benign and malignant tumours. Young women with NF1 (<50 years) have an up to five-fold increased risk of breast cancer. The UK adopts moderate cancer risk guidelines of NICE, advising women with NF1 to attend breast screening from 40 years. Previous results from a systematic review and meta-analysis found that breast cancer in this cohort predominantly occurs from 34 to 44 years. Without earlier screening, breast awareness is fundamental. Reflexive thematic analysis and semi-structured interview questions based on the health belief model explored whether a tailor-made breast cancer awareness intervention would be beneficial by examining beliefs, screening attitudes and breast cancer awareness of young women with NF1. Findings suggest the establishment of accessible and accurate NF1 and breast awareness information, development and implementation of a breast awareness intervention for young women with NF1, and healthcare professionals.

## Introduction

### Neurofibromatosis type 1

Neurofibromatosis type 1 (NF1) is a genetic disorder with a prevalence of 1 in 2500–3000 individuals worldwide (Cieza Rivera et al., 2024). Common diagnostic features include café-au-lait macules, freckling, optic pathway gliomas (Santiago-Cruz et al., 2024) and neurofibromas which are peripheral nerve sheath tumours (Dischinger et al., 2018; Magro et al., 2022). Approximately 50% of cases are due to spontaneous or ‘de novo’ mutation, where no family history of the disease is present (Carton et al., 2023; Hirbe and Gutmann, 2014). NF1 varies in rate of progression, disease severity and expressivity, creating management challenges (Uusitalo et al., 2015).

Mutations within the NF1 gene, which is deemed a tumour suppressor (Da Silva et al., 2015), instigates the condition. This gene has one of the highest spontaneous mutation rates within the human genome (Boyd, 2009), with neurofibromin loss resulting in uncontrollable cell proliferation (Madanikia et al., 2012). This predisposes individuals with NF1 to a range of benign and malignant tumour types (Maani et al., 2019; Uusitalo et al., 2016) including gastrointestinal, ovarian, bone and breast cancers (Landry et al., 2021; Seminog and Goldacre, 2013).

### NF1 and breast cancer

Historically, uncertainty surrounded the link between an elevated breast cancer risk and NF1, with death certificates, for example, failing to show that a woman had NF1 when they died of breast cancer (Evans et al., 2011). However, recent research has established a strong link (Dischinger et al., 2018; Evans et al., 2020; Maani et al., 2019; Uusitalo et al., 2017). While women with NF1 over 50 years of age do not differ significantly in breast cancer risk compared to women in the general population (Evans, 2012; Uusitalo et al., 2016), those younger than 50 years have an up to five-fold increased risk (Maani et al., 2019). Results from a systematic review and meta-analysis found that this occurrence predominantly presents in women aged 34–44 years (Suarez-Kelly et al., 2019), with one theory suggesting that individuals with NF1 experience gene mutations that accumulate causing malignancy to occur earlier (Uusitalo et al., 2017).

Breast cancer in this cohort has a higher frequency of grade 3 (Yap et al., 2018) and larger tumour size compared to non-NF1 breast cancers (Uusitalo et al., 2017), with later presentations resulting in a poorer prognosis (Sheridan et al., 2014). Breast cancer awareness and beliefs, including knowledge of what changes to be aware of and the increased risk, may encourage earlier presentations, resulting in earlier diagnoses and treatment for an improved survival rate from breast cancer (Ginsburg et al., 2020; Linsell et al., 2010; O’Mahony et al., 2017).

However, NF1 knowledge limitations have been found in varying geographical locations. Karwacki (2020) reported significantly limited knowledge about NF1 related breast cancer risk, with 68% of polish women with NF1 not receiving such information. Similarly, only a limited number of participants within an Australian cohort had a good understanding of NF1, and its related complications and risks, with many not pursuing appropriate medical advice (Oates et al., 2013). Brazilian participants with NF1 also lacked sufficient NF1 knowledge, with some unaware of the associated and increased predisposition to cancers (Bicudo et al., 2016). Subsequently, a lack of NF1 specific breast awareness may contribute to delayed diagnoses if the woman and/or healthcare professional assume a lump is a benign neurofibroma (Evans et al., 2011).

Women with NF1 are at a moderate risk of breast cancer (Sharif et al., 2007), therefore, the United Kingdom follows the recommendations of the National Institute for Health and Care Excellence’s (2023) guidance that women with NF1 attend annual mammograms from the age of 40 years. However, a lack of earlier breast screening guidelines awareness among young women with NF1 has been previously found (Fleming et al., 2023) which may subsequently affect screening attitudes. Despite these guidelines, earlier screening has also been recommended for this cohort (Carton et al., 2023; Evans et al., 2020), with the National Comprehensive Cancer Network (2017) of North America, the NSW Cancer Institute of Australia (2021) and the Protocole National de Diagnostic de Soins (mandatory French clinical practice guidelines for rare diseases; Bergqvist et al., 2020), recommending screening from 30 years of age. Without earlier screening, the first line of defence for these women is breast awareness with a positive screening attitude to attend screening when available to improve the prognosis of those who are diagnosed with breast cancer.

### Objectives

Breast cancer awareness interventions aim to increase both knowledge and screening uptake (Anastasi and Lusher, 2017), potentially reducing mortality rates. However, this area is under researched within an NF1 context. A search of the EBSCOhost database including MEDLINE, APA PsycArticles, APA Psychinfo on 23rd October 2022 (again on 6th March 2024), with predetermined Boolean phrases within the database fields of ‘neurofibromatosis type 1’ (top field) AND ‘breast cancer awareness’ (field 2) AND ‘ “breast screening” or “mammogram” or “mammography” ’ (field 3) returned no results. When field 3 was removed, only one study (Karwacki, 2020) was returned highlighting a lack of breast cancer awareness among Polish women with NF1. To the best of the PI’s knowledge, there were no known breast awareness interventions for young women with NF1 at the time of conducting this research in 2020/21.

An exploration into beliefs, screening attitudes and breast cancer awareness of young women with neurofibromatosis type 1 will aid in ascertaining whether a tailormade intervention would be of benefit, with findings contributing to its development if deemed necessary.

This study therefore asks the research question: What are the beliefs, screening attitudes and breast cancer awareness of young women with neurofibromatosis type 1?

## Methods

### Ethical statement

This study received ethical approval from Manchester Metropolitan University’s ethics review committee (Approval: EthOS 28300) on 11th March 2021 to undertake interviews. Participants were emailed a consent form for familiarisation the day before interviews, with all participants providing verbal informed consent audio recorded prior to enrolment in the study and commencement of interviews. Participants were referred to by pseudonym, and names anonymised by a unique six-character participant ID. Due to restrictions of face-to-face research occurring during periods of the COVID-19 pandemic, data collection took place via Microsoft Teams and by telephone.

### Design

A qualitative design allows for exploration of meanings, recognising that there is no single truth whereby subjectivity of individual beliefs and attitudes is incorporated into the analysis (Braun and Clarke, 2013). Both factual breast cancer awareness and breast cancer screening information are freely available in society throughout the United Kingdom, however individual attitudes and beliefs vary in context within society and within women with NF1. The Principal Investigator (PI) recognises her own presence within this analysis as a woman who had breast cancer at 35 years of age, and volunteers for a cancer charity as a peer support and awareness advocate. Recognising that the subjectivity within this research may be socially influenced, and that the PI can only partially access these beliefs and attitudes because of differing contexts, an ontological critical realist position with a contextualist epistemological assumption was adopted (Tebes, 2005).

Acknowledging both the participants’ and PI’s positions, reflexive thematic analysis was employed, recognising the PI’s active role in knowledge production (Braun and Clarke, 2019).

### Participants and recruitment

A homogeneous purposeful sampling technique to identify common patterns shared (Palinkas et al., 2015) was used to recruit young women with NF1. Participants were recruited with permission through the Childhood Tumour Trust, a charity based in England that helps individuals and their families affected by NF1. The charity founder acted as gatekeeper. The research’s objective, and inclusion criteria were advertised through the charity’s website and social media platforms of X, Facebook and Instagram over a 4-week period.

As annual breast cancer screening is available to women with NF1 from 40 years of age, the inclusion criteria research included participants:

- female with NF1 aged 18–40 years.
- with access to either a telephone or technology that facilitated Microsoft Teams for interviews.

Exclusion criteria included participants:

- with a history of breast cancer.
- over 40 years of age attending annual breast screening.
- not possessing a satisfactory comprehension of English as no funding was available for translation.

Thematic analysis studies often cite ‘data saturation’ for sampling size as a point when no additional data is found (Morse, 2015). However, Braun and Clarke (2020) argue that potentially new themes can always be realised. Malterud et al. (2016) reason that reaching ‘information power’, a conclusion of topic relevance, may provide better consideration. In this instance, information power was achieved from 10 completed interviews and recruitment was ceased. Throughout the recruitment process, only one participant became unavailable for interview.

### Data collection

Interview data from 10 semi-structured one-to-one interviews were collected via telephone/Microsoft Teams due to Covid-19 restrictions and recorded by Dictaphone from 1st April to 18th May 2021, ranging from 19 to 37 minutes in length. Overall, sound quality was good to allow transcription. Questions were grounded within the health belief model (Rosenstock, 1966) to maintain the research exploration of screening attitudes, awareness and beliefs with questions built on perception constructs of susceptibility, severity, benefits and barriers.

Examples of questions are, ‘What is your understanding of being “breast aware”?’ (perceived susceptibility), ‘how often do you check your breasts?’ (perceived severity), ‘what do you think stops women with NF1 seeking help if they think something is not right with their breast?’ (perceived barriers) and ‘what do you think, if any, are the benefits of being breast aware?’ (perceived benefits).

### Methods to ensure rigour

While understanding the subjectivity of qualitative analysis, this does not translate to research lacking rigour. The *Critical Appraisal Skills Programme* (CASP, 2021) checklist considering results, validity and research value was referred to throughout. Rigour within qualitative analysis comprises of credibility, transferability, dependability and confirmability (Lincoln and Guba, 1985). A thorough methods section has been provided to allow for transferability (Thomas and Magilvy, 2011). With reflexivity aiding in credibility, reflective logs were written after each interview to reduce misinterpretation of participants’ experiences (Krefting, 1991). Reflective practice also provides information of research conduct, providing confirmability within the research conduct (Johns, 2009).

Member checking, whereby analysis is checked with participants to ensure correct interpretations and credibility (Lincoln and Guba, 1985) was not conducted. Reflexive thematic analysis incorporates the PI’s position throughout, adopting an ontological critical realist position, recognising both truth and interpretation. Member checking works within a realist framework of seeking participants’ truth and may therefore conflict with both method and ontological position (Braun and Clarke, 2013). The use of coding books enhances dependability (Thomas and Magilvy, 2011) and credibility (Smith and Firth, 2011), however reflexive thematic analysis represents PI interpretations, therefore it is discouraged to attempt to provide accurate coding accounts, or consensus among others (Byrne, 2022). However, the use of the health belief model within interview question design increases dependability by maintaining a focus on the aim and participants’ experiences.

### Data analysis

No software was employed for analysis. Initially the PI became familiar with the data by listening and transcribing orthographically. Familiarisation continued by reading and re-reading through the transcripts, identifying information relevant to the research question. Preliminary notes highlighted initial observations. Reflective logs were also referred to, to refresh interview memory.

Phase 2 generated short descriptive labels of initial codes to inform of commonalities within the analysis (Braun et al., 2016). Within reflexive thematic analysis, the coding process is organic with no coding framework (Braun and Clarke, 2020). It is also iterative, with coding evolving during data re-familiarisations (Braun and Clarke, 2014). Both semantic coding identifying explicit meaning, and latent coding attempting to ascertain underlying assumptions were adopted (Byrne, 2022). Examples of codes are ‘lack of knowledge (woman)’, and ‘lack of knowledge (Health Professional)’.

The analysis progressed from individual interview coding to shared meanings across the dataset to create candidate themes. This is an active process within reflexive thematic analysis, whereby the PI interprets code relationships when forming themes (Byrne, 2022), however the focus remains on the research question (Braun and Clarke, 2013). Candidate themes were formed several times with the PI having to ‘let go’ of those that were not adequate (Braun and Clarke, 2013).

One theme and four subthemes communicate the research findings with names chosen to lucidly capture the dataset meanings (Braun and Clarke, 2013). Data extracts were utilised illustratively, with the analytic narrative providing a description and interpretation of the theme (Braun and Clarke, 2013). ‘Cleaning up’ was performed on data extracts (Braun and Clarke, 2013) whereby hesitations and repetitions, for example the excessive use of ‘like’ within sentences, were deleted.

## Results and Discussion

Despite each facet of the health belief model (Rosenstock, 1966) employed within interview questions to address the research question, perceived information barriers are observed as having a profound effect on beliefs, attitudes and awareness. It is therefore recognised as a theme *the metastasis of malignant information barriers*, hierarchically above four subthemes.

### Theme: The metastasis of malignant information barriers

This theme refers to the outcomes of perceived information barriers as ‘malignant’, spreading to affect areas of interest within this research. These are expanded upon within the subthemes but are recognised as originating from both healthcare professionals and the women themselves.

Healthcare professionals have been identified as having a low level of rare disease information (Kole and Faurisson, 2009; Miteva et al., 2011), potentially hindering an ability to recognise an increased breast cancer risk with NF1. This is communicated across all interviews, for example:

Willow: *Whenever I’ve been to A and E, and they ask about any previous medical conditions, my mum will be, ‘yeah she’s got neurofibromatosis type 1’, and they’ve gone, ‘what’s that?’*

This knowledge insufficiency also includes healthcare professionals that construct assumptions that medical complaints of women with NF1, are a causality of NF1. Medical training may at times rely on recalling biomedical theory, rather than adopting holistic and critical thinking (Langberg et al., 2019). Conversely, a biopsychosocial approach (Engel, 1980) incorporates a patient perspective with psychological and social facets. Anxiety, frustration and self-doubt are experienced by those that encounter healthcare professional knowledge insufficiency (Vandeborne et al., 2019), with each participant experiencing such emotions. Jay communicates frustrations of a biomedical approach:

Jay: *[…] I’m just sick to death of everyone saying NF is the reason that this is happening. It’s NF, it must be NF. […] I’d rather if people assess the situation, instead of just assume straight away, it’s because of the NF.*

Perceived information barriers also derive from the women attempting to fill these information voids. This subsequently affects beliefs and attitudes surrounding breast cancer awareness, and screening, with the consequences of this information seeking explored in greater detail within the ensuing subthemes.

### Subthemes

#### Fitting pieces together down rabbit holes

With limited educational support available, the women interviewed are driven to find information themselves, like Sophie:

Sophie: *[…] you don’t know where to start, and then you go to the GP or the health professional for support and then they don’t know, so then you know, you’re like ‘well where do I turn?’, ‘who do I turn to?’, ‘where do I get the information from?’ and that’s why I started doing my own research because I didn’t know where to go or what to do or who could help me.*

It becomes a process of sense making. Constance notes, ‘*it’s fitting all the pieces together myself*’. While the women indicate evidence-based sources such as cancer and tumour charities, there is a profound reliance on social media and social networking sites. The latter have become influential health platforms for knowledge acquisition (Capurro et al., 2014; Litzkendorf et al., 2016), health literacy and self-efficacy (Dredze et al., 2016; McGloin and Eslami, 2015), often providing an opportunity for the phenomenon of serendipity (Erdelez, 1999) whereby individuals find coincidental information pertaining to their condition:

Ariel: *[…] it was only by chance, like pure chance that I saw it […] I didn’t know about breast cancer awareness specifically for NF1, I knew about the tumours, from a certain age that they can turn malignant, but I never really put the two and two together, like the breast cancer.*

However, this environment creates learning complexities (Biancovilli et al., 2021), with misinformation and confusion (Barua et al., 2020; Wang et al., 2019). The process can become a burden, ‘*[…] it’s like how far down the rabbit hole do I want to go? […]*’ (Ariel). The women also convey incidences of healthcare professionals using the Internet for information, with Teddy remarking:

Teddy: *One thing that stops me from wanting to go to the doctors in general for anything, any problems, is doctors not having enough knowledge […] they’ll sit and google things in front of you about neurofibromatosis […]*

Domaradzki and Walkowiak’s (2021) research found that medical, nursing and physiotherapy students report the Internet as the most important resource for rare diseases. Depending on the source, there can be a prevalence of health misinformation online (Suarez-Lledo and Alvarez-Galvez, 2021). Furthermore, despite the acknowledged relationship between NF1 and breast cancer, the women also report a lack of accessible and evidence-based information within reputable sources such as cancer charities:

Teagan: *[…] there’s no information there saying that if you have NF, you are at higher risk, but there are other things on there if you have the BRCA gene, so it’s very much a one-way street where I think we kind of get forgotten about […]*

Information ambiguity may explain differing breast examination behaviours varying from every day to every couple of months, and perceived susceptibility and severity to breast cancer from, ‘*[…] I believe it’s only slightly more than someone without NF*’ (Constance), to, ‘*[…] I feel like the chances are quite high […]*’ (Hermione).

#### The battle to be taken seriously

Healthcare professionals’ delivery of positive cognitive and emotional care involves relationship building, comprising the patient’s perspective, providing information, mutual agreement and shared decision making (Riedl and Schü ßler, 2017). However, for many interviewed, encounters with healthcare professionals are challenging with healthcare professionals’ perceived knowledge deficiency translating to an unsupportive attitude, for example:

Sophie: *[…] if the person that they are going to go and ask for help has a lack of knowledge, they’re not going to be reassured, and they are not going to get supported in the correct manner. It becomes that battle to be taken seriously […]*

Healthcare professionals that exhibit overconfidence bias, the belief that one knows more than is true (Croskerry, 2003), is associated with reduced quality of care (Pisklakov et al., 2014), misdiagnoses and a lack of understanding of a patient’s perspective (Domaradzki and Walkowiak, 2021). Snow et al. (2013) identify that some healthcare professionals consider patients that challenge decisions as non-compliant. Within the interviews, women communicate these experiences:

Cooper: *[…] it was very like the doctors were like ‘oh it’s not that, it’s fine’. I was like, ‘no but you need to check’. They were really standoffish.*

Challenging attitudes from healthcare professionals negatively affect the women’s emotions, and their trust in these individuals as depicted in Teddy’s comment:

Teddy: *It makes you feel, quite like low, and it makes you feel a bit insecure and unsafe with that doctor because it’s like they don’t know what they’re doing.*

While it is understandable that healthcare professionals cannot be privy to all information on NF1 and its associated breast cancer risk, a collaborative doctor-patient partnership is important within medical uncertainty (Kornelsen et al., 2016), with open communication rated by patients as the most important aspect in developing these relationships (Schmidt et al., 2012).

#### Fighting your corner within blurred lines

Seven participants had a good understanding of the general signs and symptoms of breast cancer, with three reporting a single indicator of a lump. Every participant indicated benefits of being breast aware, with alleviating anxiety, ‘*[…] it calms my anxiety down to make sure that the lumps that are there are not getting bigger*’ (Hope), and early detection for better prognosis, ‘*it’s better to deal with instead of later on down the line. You could have saved a situation*’ (Jay), being frequently cited.

A common cue to action to being breast aware and seeking help is observed in those that have experienced breast cancer through a friend or family member’s diagnosis. Rosenstock (1974) notes that this is important in prompting behaviour, as conveyed by Hermione:

Hermione: *I know people on my grandma’s side has had it, so I feel like that’s made me a bit more cautious because it’s been in the family, and now especially because I’ve got NF1, I’ve got a bit more of a higher chance, that’s made me even more like aware of it, so I don’t think I’d even think about. I’d just phone them, even if it’s just a peace of mind whether they can tell me whether it is a fibroma or whether I do need to go and like get it properly checked.*

Being breast aware empowers these women to take control, as captured in Ariel’s comment, ‘*I think knowing what’s normal for you and just having that confidence to know that something’s not right, so then if you do go to the GP, you can like fight your corner […]*’. Developing awareness at a younger age enables self-efficacy as rationalised by many of the women. For example:

Teagan: *[…] you start to know your body so if you check at 16 and you think, okay there’s a fibroma there, and you go down ten years down the line, that’s a fibroma, but that one’s not, well then, they can just know and go to the doctor and be, ‘right, this is not a fibroma, this is something new. I want this to be checked out’.*

Self-efficacy enables confidence in initiating and maintaining behavioural change of checking breasts and being breast aware (Moey et al., 2021). Individuals high in self-efficacy when conducting self-breast examination, have a higher rate of detecting tumours successfully and correctly (Khiyali et al., 2017). However, a dominant barrier perceived throughout the interviews was uncertainty in knowing what exactly to be aware of within the context of NF1, such as Ariel’s observations:

Ariel: *[…] I don’t know what I’m actually checking for. I’m like ‘well is this normal?’, and it’s like that’s normal to me, but is that normal for everybody else and should I be getting these checked? And I think that’s where it’s a blurred line for me because I don’t want to waste the GP’s time especially with Covid and everything. Like they’re under so much pressure.*

Tavafian et al. (2009) find that perceived barriers are lower in those that have a high self-efficacy in breast examination. With a lack of specific breast awareness educational resources for NF1, the women continue to experience doubt, which may impede their motivation to check their breasts (Didarloo et al., 2017).

#### Exposed but no naked truths

None of the women interviewed are at an age where they attend regular breast screening, yet it is acknowledged that screening will play an important role in detecting cancer early (Bonilla et al., 2017). It is crucial that these women attend screening when it is available, and therefore screening beliefs and attitudes are explored. The screening benefit, ‘*to make sure everything’s okay and nothing’s there that shouldn’t be*’ (Willow) is essentially shared amongst the women, similar to the general population’s consensus (Chorley et al., 2018). However, potential future screening attendance barriers specific to women with NF1 are highlighted within the interviews such as a radiation risk of generating tumours (Suarez-Kelly et al., 2019), and overdiagnosis:

Sophie: *I’m a bit on the fence as to whether it’s a good idea or a bad idea. At a young age obviously the exposure to radiation, or the overdiagnosis that you get, with breast cancer and the treatment that follows, that makes me a bit anxious because there are other options available, like ultrasound […]*

Psychological issues can negatively impact on future screening attendance (Nelson et al., 2016). Being self-conscious is also identified by the women, ‘*women with all the lumps, all the tumours might be a bit more self- conscious to actually go and expose their condition to a stranger*’ (Hope), with this self-consciousness particularly experienced by patients with NF1 (Granström et al., 2012). Self-consciousness is also discussed in relation to the opposite sex performing examinations, ‘*[…] there’s a lot of male practitioners doing it and I think there’s not enough female doctors or nurses doing it. I think that can stop people*’ (Cooper). However, some recognise screening benefits as outweighing psychological discomforts, ‘*[…] it’s nothing to be embarrassed about, it should just be, get yourself checked*’ (Jay).

Preliminary findings from Crook et al. (2021) indicate that most women with NF1 do not experience adverse consequences attributable to breast screening, with the majority being satisfied with regular surveillance.

Only one participant was aware that breast screening occurs from the age of 40 years for women with NF1 in the United Kingdom. Confusion was evident, with some stating access from 30 years which may come from their independent research on screening information that is applicable to other countries. Others were not aware of earlier screening compared to the general population, ‘*I didn’t know that we was entitled to have that, ‘cos no one’s really said anything about it*’ (Hermione). Healthcare professionals’ perceived lack of awareness also creates screening barriers for women with NF1:

Teagan: *[…] they were saying they were trying to get a scan and I think the doctor just wasn’t aware of NF of having higher percentages, so he was just like, ‘no, have it when it’s, when everybody else has it’.*

While screening programmes are aimed at detecting cancer early allowing for more effective treatment (Cortesi et al., 2019), without the correct information available, women with NF1 may miss important opportunities to get screened.

## Conclusion

This research aimed to examine the beliefs, screening attitudes and breast cancer awareness of young women with NF1 by semi-structured interviews based on the health belief model (Rosenstock, 1966). This was to ascertain whether a tailor-made intervention may be beneficial in enhancing breast awareness behaviours among women with NF1. Addressing confusions surrounding perceived susceptibility and severity with risk, communicating the earlier screening recommendation from 40 years of age, and of what to be aware of specifically with NF1 would be beneficial. This is concurrent to Anastasi and Lusher’s (2017) findings that such interventions aid in increasing awareness and screening uptake. However, this study subsequently identified that the research aim was too narrow. Within the reflexive thematic analysis, it was also identified that wider perceived information barriers exist and influence health belief model components. These subsequently have a profound effect on beliefs, attitudes and awareness. Findings from the interviews within subthemes communicate confusion experienced by women and healthcare professionals when searching NF1 and breast cancer risk information. Other important factors have also been highlighted, particularly healthcare professionals’ perceived challenging attitudes that subsequently affect professional-patient relationships and help-seeking behaviours. These culminate to form breast awareness barriers among women with NF1, and create self-efficacy doubt for self-breast examination, and confusion regarding breast screening eligibility, potentially affecting future screening attendance.

These results are worthy, considering that as stated in the introduction, the link between NF1 and increased breast cancer risk has only been firmly established in recent years with no known breast awareness interventions for young women with NF1 at the time of conducting this research in 2020/21. Findings will contribute to the design of a tailormade NF1 breast awareness intervention which will be disseminated in future publications. It is proposed that future research should explore the feasibility of a breast cancer awareness intervention for young women with NF1 *and* healthcare professionals, acknowledging the importance of involving numerous stakeholders. An exploration into areas of capability, motivation and opportunity, should be explored as found in the COM-B model for behaviour change (Michie et al., 2014), to enhance clinical efficacy and patient-professional relationships.

Designing tailormade interventions for young women with NF1 to enhance breast cancer awareness is therefore not viable on its own. Awareness is a shared experience, whereby healthcare professionals’ knowledge of the condition and associated breast cancer risks is critical in aiding breast cancer awareness in women with NF1. With poor rare disease knowledge of healthcare professionals understood as also contributing a barrier to breast awareness within an NF1 context that may have further implications on accessing earlier screening, for example, this aligns with findings within rare disease literature that it is an ongoing issue with healthcare professionals not having an acceptable understanding of such conditions (Domaradzki and Walkowiak, 2021; McMullan et al., 2020; Ramalle-Gómara et al., 2020; Sanges et al., 2020). A dual approach therefore is required to enhance awareness within both parties to improve patient-professional relationships, and crucially, for better outcomes for young women with NF1 that are diagnosed with breast cancer. The following clinical recommendations are therefore recommended:

• The establishment of accessible and accurate information specific to NF1 and breast awareness
• Development and implementation of a breast awareness intervention for young women with NF1
• Development and implementation of an NF1 and breast awareness intervention for healthcare professionals

Reflexive thematic analysis’ acknowledgement of the PI’s position within the research, may have influenced aspects of findings, particularly having personally undergone treatment for breast cancer as a young woman and recognising the importance of being breast aware. However, interview questions were based on all aspects of the health belief model, including both benefits and barriers. Qualitative analysis utilises retrospective recall and is subject to unintentional omissions and errors, therefore reflective logs were written after each interview to ensure aspects were not overlooked. Nonetheless, it is recommended that future research adopts a mixed methods approach. Triangulation aids analysis accuracy (Braun and Clarke, 2013) and validity (Giles, 2014).

Despite the findings, it is important to note that healthcare professionals were not interviewed within this research. Reports pertaining to these individuals within this research were communicated by the young women with NF1 interviewed. It is recommended that future research should conduct interviews to understand reports of NF1 knowledge insufficiency and attitudes from healthcare professionals’ perspectives. Any recommendations that are to be acted upon should emanate from shared discussions amongst relevant stakeholders throughout the entire intervention design.

This is a new area of research that warrants a thorough investigation, with it prudent that a proposed tailormade breast awareness intervention for young women with NF1 is sincerely deliberated. Young women with NF1 can be better enabled to discover potential malignant changes, and healthcare professionals can be more confident in acting to conceivably increase these women’s chances of survival if diagnosed with breast cancer.
